# LINKING DISADVANTAGED HEALTHCARE PATIENTS TO HOME- AND COMMUNITY-BASED SERVICES: CAN NEEDS BE MET?

**DOI:** 10.1093/geroni/igac059.1540

**Published:** 2022-12-20

**Authors:** Farida Ejaz, Miriam Rose, Courtney Reynolds

**Affiliations:** Benjamin Rose Institute on Aging, Cleveland, Ohio, United States; Benjamin Rose Institute on Aging, Cleveland, Ohio, United States; Benjamin Rose Institute on Aging, Cleveland, Ohio, United States

## Abstract

As part of a larger study examining the social determinants of health, the current analysis focuses on 254 older and disabled, Medicare Advantage patients from 19 primary care clinics in Texas. The patients faced challenges such as depression, limitations in activities of daily living (ADL) and dementia. They received a home-based, social work intervention to examine their needs, an individualized care plan was created, they were offered home and community-based services, and followed over a four-month period. The median age of the sample was 69 years, 71% were Hispanic/Latino, 80% had a high school education or less, and 76% had a monthly income of less than $1,361. A total of 823 needs were identified in these patients, and 1,126 service recommendations were made. Some needs required more than one service recommendation or vice versa. The most frequently identified needs involved food assistance (136 patients received 220 nutritional service recommendations), home modifications/housing (118 patients offered 159 services), and ADLs (115 patients, 147 services). During the four-month period, social workers reported that services related to food assistance met patient needs 61% of the time; 52% for home modifications/housing; and 75% for ADLs. Reasons for unmet needs included service applications still in process/waiting lists; services being unavailable (e.g., lack of mental health providers); and refusals by patients, and family/friends. Practice and policy implications include the possibility that four months is not enough time to fully address needs, and some patients may need more intensive assistance and motivation to apply for and access services

